# SERS detection of the biomarker hydrogen cyanide from *Pseudomonas aeruginosa* cultures isolated from cystic fibrosis patients

**DOI:** 10.1038/srep45264

**Published:** 2017-03-28

**Authors:** Rikke Kragh Lauridsen, Lea M. Sommer, Helle Krogh Johansen, Tomas Rindzevicius, Søren Molin, Lars Jelsbak, Søren Balling Engelsen, Anja Boisen

**Affiliations:** 1 DTU Nanotech, Technical University of Denmark, Ørsteds Plads 345B, 2800 Lyngby, Denmark; 2 DTU Biosustain, Technical University of Denmark, Novo Nordisk Foundation Center for Biosustainability, Kogle Allé 6, 2970 Hørsholm, Denmark; 3 Department of Clinical Microbiology 9301, Rigshospitalet, Juliane Maries Vej 22, 2100 København Ø, Denmark; 4 DTU Systems Biology, Technical University of Denmark, Matematiktorvet 301, 2800 Lyngby, Denmark; 5 Department of Food Science, University of Copenhagen, Rolighedsvej 26, 1958 Frederiksberg C, Denmark

## Abstract

*Pseudomonas aeruginosa* is the primary cause of chronic airway infections in cystic fibrosis (CF) patients. Persistent infections are seen from the first *P. aeruginosa* culture in about 75% of young CF patients, and it is important to discover new ways to detect *P. aeruginosa* at an earlier stage. The *P. aeruginosa* biomarker hydrogen cyanide (HCN) contains a triple bond, which is utilized in this study because of the resulting characteristic C≡N peak at 2135 cm^−1^ in a Raman spectrum. The Raman signal was enhanced by surface-enhanced Raman spectroscopy (SERS) on a Au-coated SERS substrate. After long-term infection, a mutation in the patho-adaptive *lasR* gene can alter the expression of HCN, which is why it is sometimes not possible to detect HCN in the breath of chronically infected patients. Four *P. aeruginosa* reference strains and 12 clinical *P. aeruginosa* strains isolated from CF children were evaluated, and HCN was clearly detected from overnight cultures of all wild type-like isolates and half of the later isolates from the same patients. The clinical impact could be that *P. aeruginosa* infections could be detected at an earlier stage, because daily breath sampling with an immediate output could be possible with a point-of-care SERS device.

Individuals with cystic fibrosis (CF) have an inherited defect in the cystic fibrosis transmembrane conductance regulator (CFTR) gene, causing thickened, dehydrated mucus to form on mucociliary surfaces. This in turn increases the risk of e.g. airway infections. *Pseudomonas aeruginosa* lung infections are the largest threat to the wellbeing and survival of CF patients, and it has been shown for a cohort of young Danish CF patients that an estimated 75% have a persistent infection already from the first detection of *P. aeruginosa*^1^.

If the child is unable to expectorate, today’s methods consist of either a larynx swab, which is insensitive, or induced sputum (naso-laryngeal suction), which is extremely invasive. *P. aeruginosa* can be difficult to detect, especially in children and young adults without a chronic infection because they have few bacteria in their airways. Therefore more sensitive and non-invasive methods are being investigated utilizing the fact that hydrogen cyanide (HCN) is a biomarker for *P. aeruginosa*^2^,^3^,^4^. In nature, *P. aeruginosa* emits the poisonous gas HCN in order to kill competitive microorganisms. This wildtype (WT)-like behavior is also expected to occur in the lungs, at least during the initial stages of infection. To sustain a persistent colonisation and infection, adaptation is necessary. In many cases this involves mutations of different genes, so-called “patho-adaptive” genes. One of the genes that have been suggested as being “patho-adaptive” (i.e. beneficial for *P. aeruginosa* to mutate) is *lasR*^5^,^6^. The *lasR* gene is a transcriptional regulator necessary for HCN production^7^, so a mutation in the *lasR* gene can imply a change in the bacterial emission of HCN. According to the clinical CF program in Denmark, patients are seen once a month in the outpatient clinic and deliver a sputum sample coughed up from the lungs. Depending on the patient’s age and ability this is either done by expectoration or, as in most young patients, induced by a tube inserted through the nose into the larynx to provoke a cough reflex after which a sample is obtained through the tube (naso-laryngeal suction). Sputum samples are then cultured to identify pathogenic microorganisms.

Several groups have investigated HCN production by *P. aeruginosa in vitro*^3^,^8^,^9^. The drawbacks of all the applied methods are that they need extensive sample handling before measurements can be obtained, and that it can only be done in solution. To the best of our knowledge, selected ion flow tube – mass spectroscopy (SIFT-MS) is the current state-of-the-art method in terms of sensitivity and specificity^10^. However, it is also an expensive and complex procedure. The technique was employed in the study by Enderby *et al*.^2^, where median values of 13.5 ppb HCN were detected directly in the breath of CF children with a newly discovered *P. aeruginosa* lung colonization^2^, whereas in the more recent study by Gilchrist *et al*.^10^, HCN concentrations at or below 7.5 ppb were detected from breath samples collected in bags and measured within 24 hours^10^. An optimal sensor would be a cheap and simple point-of-care device, allowing for measurements to be carried out directly on human breath, without pre-handling of the samples. The so-called surface-enhanced Raman scattering (SERS) technique can potentially fulfill these requirements since hand-held Raman spectrometers are progressively becoming cheaper, better and smaller^11^.

Raman spectroscopy is a fingerprinting technique that measures the inelastic scattering of monochromatic light when interacting with matter. The frequency shift (Raman shift) of the inelastically scattered light corresponds to the energies of the molecular vibrations^12^. HCN contains a triple bond between C and N, which will result in a Raman band around 2135 wavenumbers (cm^−1^). But since the inelastically scattered light is only a tiny part of light scattered from a sample (<10^−8^), Raman spectroscopy in general has a weak signal-to-noise ratio, and there is a need for signal enhancement. Using SERS, the Raman signal of small molecules is enhanced by utilizing the collective oscillations of conducting electrons (surface plasmons) that take place in the vicinity of clustered noble-metal nanoparticles^13^,^14^. SERS has been used in a wide array of medical applications^15^,^16^,^17^. An example hereof is using label free Ag colloid SERS to detect single-nucleotide mismatch in short DNA sequences to discover even small nucleotide changes^18^. We have demonstrated previously that the SERS technique is suitable for cyanide quantification with the estimated lower limit of detection between 1.8 and 18 ppb using highly sensitive gold-coated silicon (Si) nanopillar SERS substrates^19^. These substrates consist of vertically standing Si nanopillars with gold (Au) caps which can lean against each other for enhanced SERS detection in liquid or gas phase^20^. In this study we utilize identical SERS substrates and investigate the capability of the method to detect the biomarker HCN(g) in emissions from clinical early and late *P. aeruginosa* isolates. In order to determine its applicability as a non-invasive diagnostic tool for detection of *P. aeruginosa* we qualitatively evaluate results which indicate HCN presence in gases from early *P. aeruginosa* isolates, and since our SERS substrate is able to detect ppb values of HCN, this novel method is expected to be able to discover *P. aeruginosa* from breath at an earlier stage than by using today’s clinical practice.

## Materials and Methods

### Preparation of the SERS substrate

**Figure 1:**
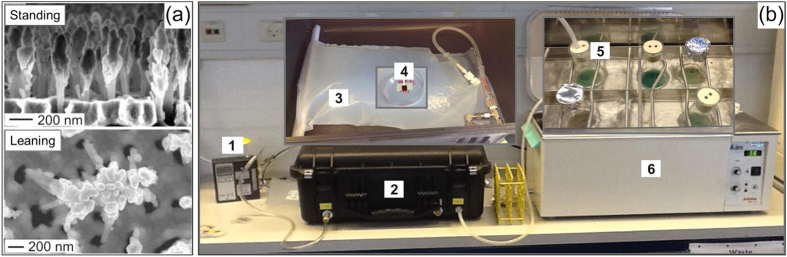
Setup for SERS measurements on bacterial volatiles. (**a**) SEM images of the SERS substrate before and after leaning for formation of electromagnetic “hot spots” for SERS enhancement. (Courtesy of Kaiyu Wu. Reprinted with permission from editor of ref. 19). (**b**) The pump (1) induces a vacuum inside the vacuum chamber (2), leading to inflation of the bag (3) and exposure of the SERS substrate (4) to volatiles created by the bacterial culture (5) in the water bath (6).

The disposable SERS substrate was etched in a single-side polished Si wafer using 3 min Reactive Ion (plasma) Etching with alternating SF_6_ bombardment and O_2_ protection of the emerging nanopillars, followed by a 1 min O_2_ cleaning step. Before use, Au was evaporated onto the Si nanopillars, forming 225 nm caps used for SERS detection. See Fig. 1(a). On the day of exposure the wafer was cut into 6 × 6 mm chips using a diamond cutter and tweezers. The chips were cleaned by immersion into ethanol (Absolute grade, CHROMASOLV R, Sigma-Aldrich) for 3 min followed by H_2_O (Molecular Biology Reagent grade, Sigma-Aldrich) for 3 min and left to dry on a tissue whereby the pillars would lean to enable SERS detection. For easy handling and transportation, the SERS substrate was mounted inside a small Petri dish, using double sided adhesive tape (Scotch), with a Post-It on top, the tacky side facing up for holding the substrate (Fig. 1(b. The wall of the Petri dish protected the delicate SERS substrate so it would not get in contact with the bag and be scratched (See section on SERS substrate exposure to bacterial emissions).

### *P. aeruginosa* reference strains

PAO1 is a reference strain commonly used to benchmark various *P. aeruginosa* strains against. PA-SD2 is a PAO1 isolate with a knockout mutation in *lasR*, as described in ref. 20. DK02 is a *P. aeruginosa* lineage that spread among Danish CF patients for over 40 years. The earliest isolate is called DK02-1973, and its *lasR* mutated strain DK02-1979^21^. All four reference strains were evaluated in the study.

### The children strain collection

At the CF clinic at Rigshospitalet in Copenhagen a special collection of bacterial strains has been sampled from CF children and young adults since 2004, resulting in a unique collection for genomics and other evolutional studies to be made^5^,^22^. From this collection early (*lasR* WT) and late (*lasR* mutant) isolates from five patients have been selected, encompassing three different clone types, for comparison of adaptations between and within patients. The selected strains originate from CF patients who today are all chronically infected, and the late isolates represent various locations of *lasR* mutations. Selected strains include the first *P. aeruginosa* isolate and at least one later isolate from the same patient after onset of chronicity and with detected mutations in the *lasR* gene. From the children strain collection, 12 *P. aeruginosa* isolates were evaluated.

### Ethics

The local ethics committee at the Capital Region of Denmark Region Hovedstaden approved the use of the samples: registration number H-4-2015-FSP. All patients have given informed consent. For patients below 18 years of age, informed consent was obtained from their parents. The study was carried out in accordance with the approved guidelines and the University Hospital Rigshospitalet approved the experimental protocol.

### Identification of mutations in *lasR*

Using a modified version of the pipeline previously described in Andersen *et al*.^23^, we identified mutations in the CF isolates using the sequences of isolates from young CF patients, previously published by Marvig *et al*. (2015)^5^. Briefly: Reads were mapped to the *lasR* reference sequence (NP_250121.1) from the reference strain *Pseudomonas aeruginosa* PAO1, using the Burrows-Wheeler alignment tool^24^ (BWA version 0.7.12), with the paired-end reads setting. Alignments were filtered to remove unmapped reads, sorted, and indexed using SAMtools^25^. Each isolate was assigned to a read group using Picard Tools (open-source software version 1.14, http://pickard.sourceforge.net) and differences between isolates and the reference *lasR* sequence were identified with SAMtools. Mutations were manually checked using the Integrative Genomics Viewer^26^ (IGV, version 2.3.68). The functional impact (missense/nonsense/silent) was determined using the translated sequence of *lasR* in the IGV. The full pipeline can be found in Supplementary material.

### Preparation of bacterial cultures

*P. aeruginosa* strain PAO1, its engineered *lasR* mutant PA-SD2 and two isolates from the classic clinical lineage DK02 from 1973 and 1979^27^, as well as *P. aeruginosa* strains from five paediatric CF patients were stored as pure cultures at −80 °C, wherefrom they were streaked onto LB agar plates and incubated at 37 °C over night (ON). One colony was then inoculated in 10 mL LB growth medium (4% salt concentration) and incubated shaking at an angle of ~45°, 225 rpm overnight ON. One mL of the ON culture was added to 9 mL of fresh LB in a 100 mL Erlenmeyer flask, plugged with a two-holed rubber plug, covered with aluminum foil and placed in a 37 °C water bath at ~200 rpm ON, wherefrom SERS substrates were exposed (Fig. 1). One flask was prepared for each measurement needed.

### SERS substrate exposure to bacterial emissions

In the setup we used an SK 224-PCMTX4 Universal air sampling pump (SKC Inc., PA, US), connected via a silicone tube to a large Vac-U-Chamber, 231–939 (SKC Inc., PA, US), Fig. 1(b. Inside the vacuum chamber a 10 L reusable 60 μm PTFE (Teflon) sample bag (Scentroid, ON, Canada), equipped with a 1/2” compression fitting was connected to the sample inlet (3). The bag was specially designed with one end open for mounting the SERS substrate, and closed by a clamp. The sample inlet of the vacuum chamber was connected via a silicone tube to the 100 mL Erlenmeyer culture flask (5), plugged with a rubber plug with two holes, one for the tube and one for air inlet. The pump was operating at ~500 mL/min for 15 min to fill the bag, followed by 5 min holding time for further substrate exposure. Before pumping out bacterial gases, all fittings were tested by closing the vacuum chamber and turning on the pump to let in air from the lab to verify that the bag would inflate and no emissions would be lost when the bacterial culture was connected to the system.

### OD and SERS measurements

The Optical density (OD) was measured at 600 nm in a UV-1800 spectrophotometer (SHIMADZU, Kyoto, Japan), giving the absorbance as a measure of cell density. SERS measurements were recorded using a FT-Raman instrument (Bruker VERTEX 70, Bruker Optik, Ettlingen, Germany), equipped with a 1064 nm laser and an InGaAs detector. Samples were measured in a 180 degrees backscattering geometry using 32 scans at a resolution of 4 cm^−1^. Each sample was measured at 5 different positions on the substrate and the 5 spectra were averaged before subsequent data analysis.

## Results and Discussion

### SERS on PAO1 and DK02 ON culture emissions

**Figure 2:**
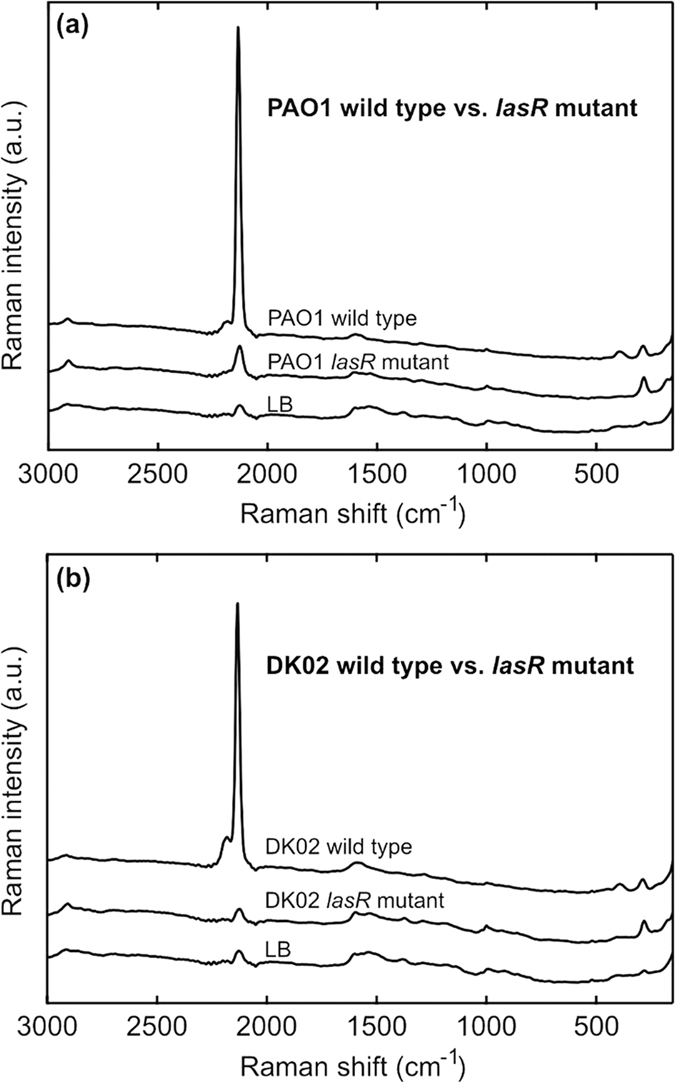
SERS on overnight cultures of PAO1, the DK02 lineage and their *lasR* mutated strains. (**a**) SERS on emissions from overnight cultures of *P. aeruginosa* PAO1, wild type vs. its engineered *lasR* mutated strain PA-SD2. (**b**) SERS on emissions from overnight cultures of the *P. aeruginosa* DK02 lineage, wild type from 1973 vs. the *lasR* mutated strain from 1979. Both wild types show intense CN signals at 2135 cm^−1^ and also a small peak at 2189 cm^−1^ due to formation of the [Au(CN)_2_]^−^ complex, whereas the *lasR* mutated strains emit no HCN. Spectra of LB emissions are included as reference.

The clinical strains were growing much slower than PAO1, and ON cultures were prepared for SERS exposure and measurements. In Fig. 2(a,b) it is seen that PAO1 and the isolate from 1973 both emitted substantial amounts of HCN, as seen in the intense C≡N stretching peak at 2135 cm^−1^, whereas the *lasR* mutated PAO1 and the late (1979) isolate did not. This was as expected since the late DK2 and the *lasR* mutated PAO1 should not have a functional LasR protein.

### HCN emission from PAO1 during growth

**Figure 3:**
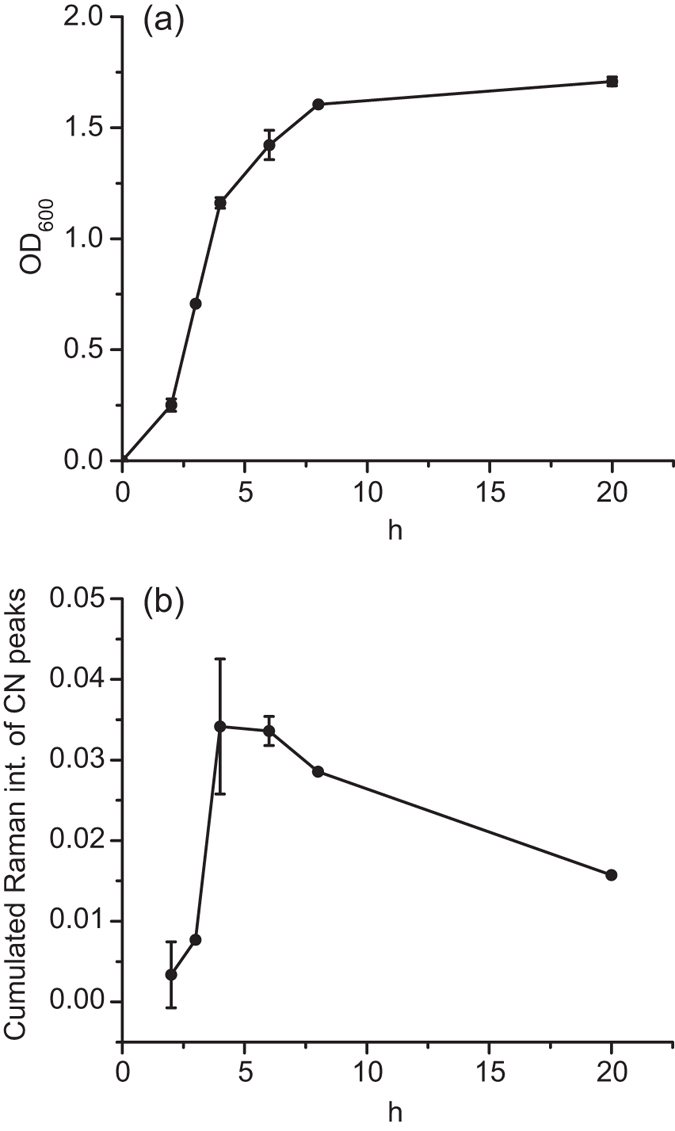
PAO1 HCN emission during growth. (**a**) Optical density (OD_600_) growth curve for PAO1 in LB medium at 37 °C. The lag phase takes about 2 hours, followed by the log phase until about 4–6 hours’ growth after which the stationary state occurs. Final OD absorbance is about 1.7 at 600 nm. (**b**) Intensity of cumulated cyanide peaks from PAO1 during growth. The cumulated Raman intensity is the (2135 + 2189) cm^−1^ SERS intensity on the PAO1 exposed substrate minus the background signal of the substrate exposed to LB emissions. It is seen that PAO1 starts producing HCN after 4 hours’ growth, which is at the end of its exponential/beginning of stationary growth phase. After 20 hours HCN is still detectable, although not as intense as after 4 hours. The shape of the CN curve resembles previous studies on P. aeruginosa HCN emission during growth^8^,^28^.

As seen in the growth curve of Fig. 3(a), PAO1 has a lag phase of about 2 hours before onset of exponential growth, with stationary growth, and expected HCN production, beginning after 4–6 hours. According to previous studies HCN production starts at the end of exponential/beginning of stationary phase^8^,^28^. This is due to the fact that HCN production is quorum sensing (QS) dependent. After a few hours on the substrate much of the C≡N peak had shifted to 2189 cm^−1^ because of sufficient amounts and the fact that it had had some time to interact with the Au-coated SERS substrate, forming the stabile [Au(CN)_2_]^−^ complex, as explained in ref. 19. Figure 3(b) shows the SERS intensity of the cumulated cyanide peaks at 2135 and 2189 cm^−1^ from 2 until 20 h (ON). It is observed that HCN production begins after 4 h growth and is still detectable in the overnight (20 h) culture, whereas the 2 and 3 h samples only had the same background as the LB reference. The results in Fig. 3(b) and the shape of the HCN emission curve with time are very similar to previous findings^8^,^29^.

### HCN emission from *P. aeruginosa* strains isolated from CF children

**Figure 4:**
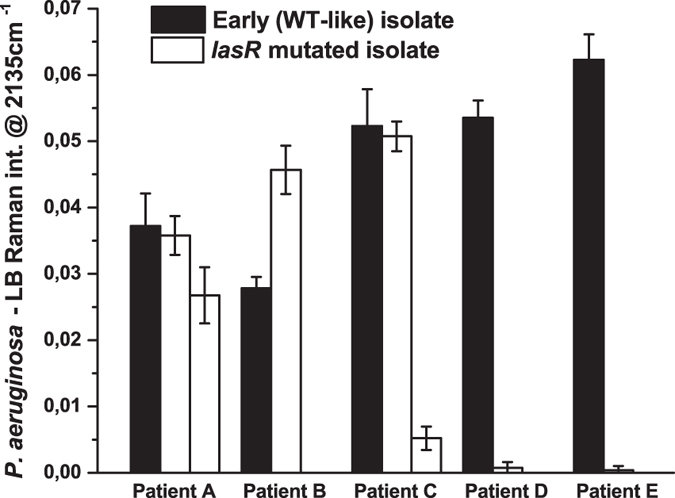
Cyanide production of clinical *P. aeruginosa* overnight cultures from 5 CF patients. The peak intensity is the SERS signal at 2135 cm^−1^ minus the background signal of the LB reference samples. It is seen that all WT-like isolates emitted clearly detectable HCN, and so did also some of the *lasR* mutated isolates. The latest isolates from patients C, D and E did not emit HCN.

A total of 12 isolates from five CF children were measured with the SERS sensor in the developed setup. In Fig. 4 the intensity of the 2135 cm^−1^ C≡N stretching band shows that all early (WT-like) strains and also some of the *lasR* mutated strains isolated from paediatric CF patients emitted HCN. Thereby, the principle had been proven that the SERS substrate was able to detect HCN from cultures of early *P. aeruginosa* colonizations, which was the aim of the study. Since it was possible to detect HCN from emissions of all early ON cultures, it was plausible that HCN could also be detected from breath of a patient with an early *P. aeruginosa* colonization. Genome sequencing of the *lasR* mutated isolates^5^ showed that isolates with *lasR* mutations located towards the 3′ terminal of the gene did not produce HCN, and it was an interesting finding that only *lasR* mutated cultures with the mutation located upstream or at the central part of the gene emitted detectable HCN. The continued production of HCN could be explained by mutations or changes in regulation of other genes that are also important for QS and HCN production circumventing a malfunctioning *lasR*.

### Clinical control strains

**Figure 5:**
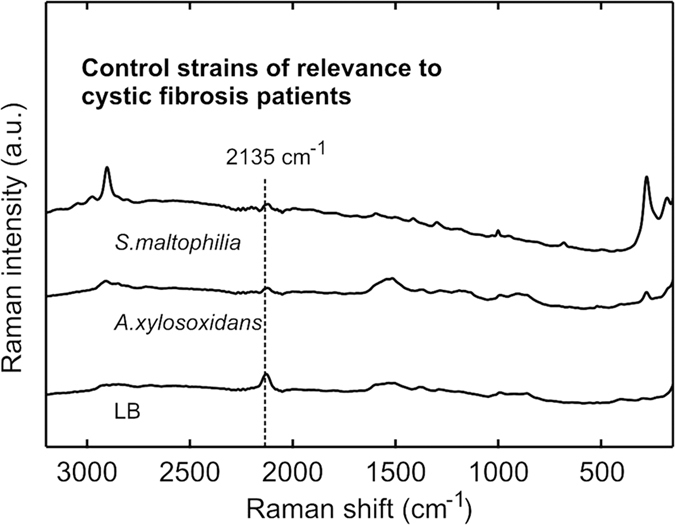
SERS on overnight cultures of *Stenotrophomonas maltophilia* and *Achromobacter xylosoxidans*. SERS on emissions from overnight cultures of *S. maltophilia* and *A. xylosoxidans.* None of the bacteria emitted any detectable HCN, as seen in the low intensity of the triple bond peak at 2135 cm^−1^. Especially the *S. maltophilia* spectrum has different peaks than *P. aeruginosa*. A spectrum of LB emissions is included as reference.

To make sure HCN could be used as a specific marker for *P. aeruginosa* colonisation, we also tested other clinically relevant species. Figure 5 shows examples of two other bacteria colonising the lungs of CF patients, and known to be able to cause chronic infections: *Stenotrophomonas maltophilia*^30^ and *Achromobacter xylosoxidans*^31^. Clinical isolates of these control strains were tested in the same setup as *P. aeruginosa*, but no HCN could be detected. The findings were in accordance with Bumunang & Babalola^32^.

## Conclusions and Outlook

We have developed a SERS-based method for detection of HCN from *P. aeruginosa* cultures, and a setup for exposure of the SERS substrate to bacterial volatiles. The purpose was to see whether the wild type like strains were producing HCN in sufficient amounts for detection by the SERS substrate and to check whether the *lasR* mutated strains were still producing HCN. For the reference strain PAO1 HCN production started at the end of stationary phase and was still detectable in overnight cultures. In all clinical wild type-like strains isolated from the airways of cystic fibrosis patients there was a clear HCN SERS signal proving the principle that we are able to detect gases from early *P. aeruginosa* isolates. HCN could only be detected from half of the strains with mutation in the *lasR* transcriptional regulator gene, which may explain why HCN cannot be detected in the breath of all patients with a chronic *P. aeruginosa* airway infection.

We acknowledge the possible limitations to this SERS-based breath device, which could have a slightly lower sensitivity than SIFT-MS, which seems to be state-of-the-art for breath detection of HCN. General limitations to ppb level breath detection could be that the child has recently been coughing, whereby the low amounts of bacterial volatiles could be lost, or that the child is too young and unable to exhale properly. Another issue could be if the infection is already chronic and *P. aeruginosa* has stopped to emit HCN. Still, our results suggest that it is indeed possible to use SERS detection of HCN as an early indicator of *P. aeruginosa* infection. The method offers the possibility of a simple, non-invasive point-of-care monitoring device, which could be used in the patients’ home or at the general practitioner for more frequent breath measurements, hopefully leading to an earlier detection of *P. aeruginosa* lung infections in patients with CF.

## Additional Information

**How to cite this article:** Lauridsen, R. K. *et al*. SERS detection of the biomarker hydrogen cyanide from *Pseudomonas aeruginosa* cultures isolated from cystic fibrosis patients. *Sci. Rep.* **7**, 45264; doi: 10.1038/srep45264 (2017).

**Publisher's note:** Springer Nature remains neutral with regard to jurisdictional claims in published maps and institutional affiliations.

## Supplementary Material

Supplementary Information
