# Supplementary material for: SERS detection of the biomarker hydrogen cyanide from Pseudomonas aeruginosa cultures isolated from cystic fibrosis patients
Source: Sci Rep. 2017 Mar 28;7:45264. doi: 10.1038/srep45264 (PMC5368648; doi:10.1038/srep45264)
Supplement: Supplementary Information [file srep45264-s1.pdf]

Supplementary material - Title page

SERS detection of the biomarker hydrogen cyanide from *Pseudomonas aeruginosa* cultures isolated from cystic fibrosis patients

Authors

Rikke Kragh Lauridsen, Lea M. Sommer, Helle Krogh Johansen, Tomas Rindzevicius, Søren Molin, Lars Jelsbak, Søren Balling Engelsen, Anja Boisen

Supplementary material

```
#!/bin/bash
ISO=$1 #Input: list of isolate names.

while read line
do

    echo "$line is being aligned with BWA (v.0.7.12) aln paired end
function (sampe)"
    bwa aln lasR.fna $line.L*_1.fq.gz > "$line"_read1.sai
    bwa aln lasR.fna $line.L*_2.fq.gz > "$line"_read2.sai
    bwa sampe lasR.fna "$line"_read1.sai "$line"_read2.sai
$line.L*_1.fq.gz $line.L*_2.fq.gz > "$line"_aligned.sam

    echo "$line is going through round one in SAMtools v.0.1.18"
    samtools view -Shb -F4 "$line"_aligned.sam > "$line"_aligned.bam
    samtools sort "$line"_aligned.bam "$line"_aligned_sort
    samtools index "$line"_aligned_sort.bam

    echo "$line read groups are added by Picard (v.1.140)"
    java -Xmx2g -jar picard-tools-1.140/picard.jar AddOrReplaceReadGroups
INPUT="$line"_aligned_sort.bam OUTPUT="$line"_aligned.RG.bam RGID=CF
RGLB=CF_LIB1 RGPL=Illumina RGPU=CF_RG1_UNIT1 RGSM=$line
VALIDATION_STRINGENCY=LENIENT

    echo "$line pileups and raw files are being produced by SAMtools
v0.1.18"
    samtools mpileup -q 20 -C50 -BDSsugf lasR.fna "$line"_aligned.RG.bam
| bcftools view -vg - > "$line"_raw.vcf
done<$ISO
```
